# Britannin suppresses MCF-7 breast cancer cell growth by inducing apoptosis and inhibiting autophagy

**DOI:** 10.22038/AJP.2023.22995

**Published:** 2024

**Authors:** Sadegh Rajabi, Mahboubeh Irani, Marzieh Moeinifard, Maryam Hamzeloo-Moghadam

**Affiliations:** 1 *Traditional Medicine and Materia Medica Research Center, Shahid Beheshti University of Medical Sciences, Tehran, Iran*; 2 *Department of Clinical Biochemistry, Faculty of Medical Sciences, Tarbiat Modares University, Tehran, Iran*; 3 *Traditional Medicine and Materia Medica Research Center and Department of Traditional Pharmacy, School of Traditional Medicine, Shahid Beheshti University of Medical Sciences, Tehran, Iran*

**Keywords:** Breast cancer, Britannin, Apoptosis, Autophagy, STAT3, JAK2

## Abstract

**Objective:**

Breast cancer is the main reason for cancer-related death in women. Britannin is a sesquiterpene lactone compound derived from *Inula aucheriana* with anti-tumor properties. We aimed to explore the impacts of britannin on apoptosis and autophagy in MCF-7 breast cancer cell line.

**Materials and Methods:**

The cytotoxic influences of britannin on MCF-7 cells were estimated by the MTT method. The expression levels of apoptosis-associated genes such as *CASP3*, *BCL2*, *BCL2L1*, *STAT3*, and *JAK2* and transcripts of autophagy markers including *ATG1*, *ATG4*, *ATG5*, *ATG7*, *ATG12*, *BECN1*, and *MAP1LC3A* were quantified using quantitative real time-PCR (qRT-PCR). Western blotting method was used to evaluate the amount of caspase 3, phosphorylated JAK2, phosphorylated STAT3, ATG1, ATG4, ATG5, Beclin1, and LC-III.

**Results:**

Treatment of MCF-7 cells with various concentrations of britannin remarkably hindered the viability of these cells compared to the controls. This compound significantly elevated the expression of pro-apoptotic caspase-3 but did not influence the levels of anti-apoptotic *BCL2* and *BCL2L1*. Britannin decreased the levels of phosphorylated forms of JAK2 and STAT3 proteins causing the blockage of the JAK/STAT pathway. Four autophagy factors expressions, including ATG4, ATG5, Beclin1, and LCIII, were reduced due to the effect of britannin on MCF-7 cells.

**Conclusion:**

Britannin triggered apoptosis in MCF-7 cells by a mechanism that led to the blockade of the JAK/STAT pathway. Moreover, britannin prohibited autophagy in these cancer cells. This may suggest britannin as an agent for the suppression of breast tumors or as an adjutant for the enhancement of anti-breast cancer drugs effect.

## Introduction

Breast cancer is the main reason for cancer-related death in women (Trayes and Cokenakes, 2021). According to the past two decades epidemiological estimates, the mortality rate of breast cancer patients is significantly increased across the globe (Azamjah et al., 2019). Breast cancer has three different subtypes that include estrogen/progesterone receptors positive, HER2 positive and triple-negative (McCarthy et al., 2021). These subtypes have specific risk profiles, and different therapeutic approaches are used for each patient depending on the subtype of tumor, disease stage, and patient preferences (Waks and Winer, 2019). Several classical therapeutic methods are available for curing patients with breast cancer, including surgery, radiotherapy, hormone therapy, and chemotherapy (Shahali et al., 2018; Waks and Winer, 2019). 

Due to the development of resistance and the occurrence of side effects, conventional methods for the treatment of breast cancer may cause disease recurrence and relapse. Therefore, it is necessary to find suitable and safer methods with minimal side effects for the patients (Malik et al., 2022). With the prompt progression in the discovery of anti-cancer remedies, molecular targets in programmed cell death pathways such as apoptosis and autophagy have gained significant attention as promising targets to suppress cancer (Ditty and Ezhilarasan, 2021; Maniam, 2021; Rahman et al., 2023). Programmed cell death is generally divided into two subcategories: apoptotic and non-apoptotic cell deaths (Chen et al., 2020). Apoptosis is promoted by caspase enzymes, but non-apoptotic cell deaths mainly occur in a caspase-independent manner (Obeng, 2021). A number of cellular signaling pathways are implicated in the promotion and progression of apoptosis. For example, several previous works have uncovered the major role of JAK/STAT signaling pathway in regulating the behavior of normal and cancer cells, and thus, blockade of this signaling pathway can stop tumor development and cause apoptotic death in many types of cancer cells (Galoczova et al., 2018; Liu et al., 2018; Xiong et al., 2008).

Autophagy is a non-apoptotic cell death process that is accompanied by a remarkable increase in the number of autophagosomes, which are vesicles with the ability to engulf intracellular organelles and transport them to lysosomes to be degraded (Jung et al., 2020). According to the literature, the role of autophagy in various types of cancer is opposing and tissue-dependent. It can be stimulated in one cancer type and prohibited in another to be utilized as a target for anti-cancer drugs (Levy et al., 2017). 

Chemical anti-cancer drugs have not markedly improved the average survival rate of patients with cancer in the past decades. Thus, the discovery of new therapeutic strategies using new and effective drugs is necessary to combat cancer. Plant-derived natural compounds, known as phytochemicals, may serve as new and potential drugs for cancer therapy (Choudhari et al., 2019; Karimi Dermani et al., 2021). Britannin is a sesquiterpene lactone isolated from *Inula* *aucheriana* with a potential anti-cancer effect on some cancer cells (Mohammadlou et al., 2022b). Our previous exploration unraveled that britannin exerts cytotoxic effects on different cancer cell lines including hepatocarcinoma, breast cancer, renal cancer, and lung cancer cell lines (Moghadam et al., 2012). Moreover, in an *in-vitro* study, we showed that britannin triggered apoptosis in two distinct breast cancer cell lines, MDA-MB-468 and MCF-7, by promoting the mitochondrial pathway of apoptosis in these cancer cells (Hamzeloo-Moghadam et al., 2015). Accordingly, in the present investigation, the effects of britannin on the expression of apoptosis and autophagy markers in MCF-7 cell line were explored. Further, the probable impact of britannin on the stimulation or inhibition of the JAK/STAT signaling was evaluated in order to identify the underlying mechanism of britannin in stimulating apoptosis in MCF-7 cells.

## Materials and Methods

**Britannin isolation protocol**

Sesquiterpene lactone britannin was derived from *Inula aucheriana* DC from the Asteraceae family. The aerial portions of the plant material were gathered from West Azerbaijan, Iran. The identity of the plant was confirmed by botanists at the Traditional Medicine and Materia Medica Research Center (TMRC), Shahid Beheshti University of Medical Sciences, Tehran, Iran. A sample (TMRC 3173) was kept at the TMRC Herbarium center. Britannin was isolated from the chloroform extract of the plant through chromatographic methods according to a previous study (Abedin et al., 2007).

**Cell culture and phytochemical compound preparation**

The human breast cancer cell line, MCF-7, was purchased from Pasture Institute, Tehran, Iran. The cancer cells were grown in Dulbecco's Modified Eagle Medium (DMEM )cell culture medium with 10% fetal bovine serum (FBS; Gibco, Germany) and penicillin/streptomycin (100 U/ml; Sigma, Germany). The cells were cultured in an incubator at 37°C in a humidified atmosphere with 5% CO_2_. Britannin was isolated and purified using a standard protocol described in our previous work (Moghadam et al., 2012). Then, a stock solution of the purified compound was prepared using Dimethyl sulfoxide (DMSO) as the solvent. 

**Cell viability assay**

The viability of MCF-7 cells was estimated by using the MTT assay method. Briefly, 7×10^3^ cells per well were cultured in 96-well microplates. Then, the cultured MCF-7 cells were treated with different concentrations of britannin (0, 1.56, 3.12, 6.25, 12.5, 25, 50, 75, and 100 μM) for 24, 48, and 72 hr. The cells in control wells were treated with the culture medium. Subsequently, the britannin-treated cells were incubated with the MTT solution (5 mg/ml) for 4 hr. Finally, the MTT solution was replaced by the DMSO solution and the number of viable cells was calculated by reading the absorbance of the formazan product at 570 nm using the spectrophotometric method. 

**Real time PCR analysis**

Real-time-PCR was used to unravel the influence of britannin on the expression of genetic markers of apoptosis and autophagy. After the treatment of MCF-7 cells with britannin (25 µM, for 48 hr), the total RNA of the treated and control cells was extracted using a RNeasy mini kit (Qiagen, Korea). Afterward, the RNA content of the samples was measured by using a Nanodrop 2000c spectrophotometer to estimate the extraction quality. The absorbance ratio of A260/A280 > 1.8 was considered to confirm the quality of RNAs. Subsequently, a cDNA Synthesis Kit (BioFact, Korea) was used to synthesize cDNA molecules from 2 μg of total RNA. Then, the alterations in the gene expression of apoptosis markers including *CASP3*, *BCL2*, *BCL2L1*, *STAT3*, and *JAK2* as well as autophagy gene markers including *ATG1*, *ATG4*, *ATG5*, *ATG7*, *ATG12*, *BECN1*, and *MAP1LC3A* were measured using ABI PRISM7900HT (Applied Biosystems, USA) qRT-PCR detection system with SYBR GREEN PCR master mix (Ampliqon, Denmark). Glyceraldehyde-3-phosphate dehydrogenase (*GAPDH*) mRNA was utilized as the internal control gene. The relative expression (fold changes) of the mentioned transcripts was calculated by 2^-ΔΔCT^ method. Table 1 shows the primer sequences utilized for the amplification of gene transcripts.

**Table 1:** Primer sequences used in the present study

**Gene name**	**Forward primer**	**Reverse primer**
***CASP3***	5′ -CTCGGTCTGGTACAGATGTCGATG-3′	5’-GGTTAACCCGGGTAAGAATGTGCA-3′
***BCL-2***	5'-CTGTGGATGACTGAGTACCT-3′	5'-GCCAGGAGAAATCAAACAGAG-3′
***BCL2L1***	5'-GCCACTTACCTGAATGACCACC-3′	5'-AACCAGCGGTTGAAGCGTTCCT-3′
***JAK2***	5'-GATGAGAATAGCCAAAGAAAACG-3′	5'-TTGCTGAATAAATCTGCGAAAT-3′
***STAT3***	5'-GCTTCCTGCAAGAGTCGAAT-3′	5'-ATTGGCTTCTCAAGATACCTG-3′
***ATG1***	5'-GTTCCAAACACCTCGGTCCT-3′	5'-CCAACTTGAGGAGATGGCGT-3′
***ATG4***	5'-AGAATGGAGTCAGTTTTATCCAAGT-3′	5'-TGGCTTTATCTGGGTGCTGG-3′
***ATG5***	5'-GCAACTCTGGATGGGATTGC-3′	5'-CAACTGTCCATCTGCAGCCA-3′
***ATG7***	5'-GAGAACATGGTGCTGGTTTCC-3′	5'-TAGGACAATCTTCGTCCTTTGAC-3′
***ATG12***	5'-TGCTGGAGGGGAAGGACTTA-3′	5'-CAGCAGGTTCCTCTGTTCCC-3′
***BECN1***	5'-GTCGCTGAAGACAGAGCGAT-3′	5'-CGATGCTCTTCACCTCGGG-3′
***MAP1LC3A***	5'-TTCCGAGTTGCTGACTGACC-3′	5'-CCCTTGTAGCGCTCGATGAT-3′
***GAPDH***	5'-ACCCACTCCTCCACCTTTGA-3′	5'-CT GTTGCTGTAGCCAAATTCGT-3′

**Western blot analysis**

To assess the impact of britannin on the levels of apoptosis- and autophagy-related proteins, the western blotting method was used. Briefly, the cultured cell line was treated with britannin (25 µM, for 48 hr), and a normal culture medium was used to treat the control cells. Then, the treated cells were harvested and lysed by using radioimmunoprecipitation assay (RIPA) buffer (Thomas Scientific Inc, USA). The protein content of the samples was determined using a bicinchoninic acid (BCA) protein assay kit (Thermo Fisher Scientific, UK), and 40 µg of total protein was isolated by the SDS-PAGE technique. The separated proteins were then transmitted onto a polyvinylidene difluoride (PVDF) membrane (Roche, Germany) and blocked with 5% non-fat milk for 1 hr at room temperature. After blocking, the blots were incubated with a dilute solution of primary antibodies (Santa Cruz Biotechnology, USA) against Caspase 3, phosphorylated JAK2, phosphorylated STAT3, ATG1, ATG4, ATG5, Beclin1, LC-III, and GAPDH proteins at 4°C overnight. Subsequently, a secondary antibody connected to horseradish peroxidase (HRP) (Abcam, USA) was applied and the protein blots were visualized through enhanced chemiluminescence (Amersham Pharmacia, Freiburg, Germany). 

**Data analysis**

All data were statistically analyzed using one-way analysis of variance (ANOVA) method followed by Duncan’s multiple test for *post hoc* comparison by GraphPad Prism software. P-values less than <0.05 were considered significant. All experiments were done in triplicate and the data are presented as mean±standard deviation (SD).

## Results

**Effect of britannin on viability of MCF-7 cells**

**Figure 1:**
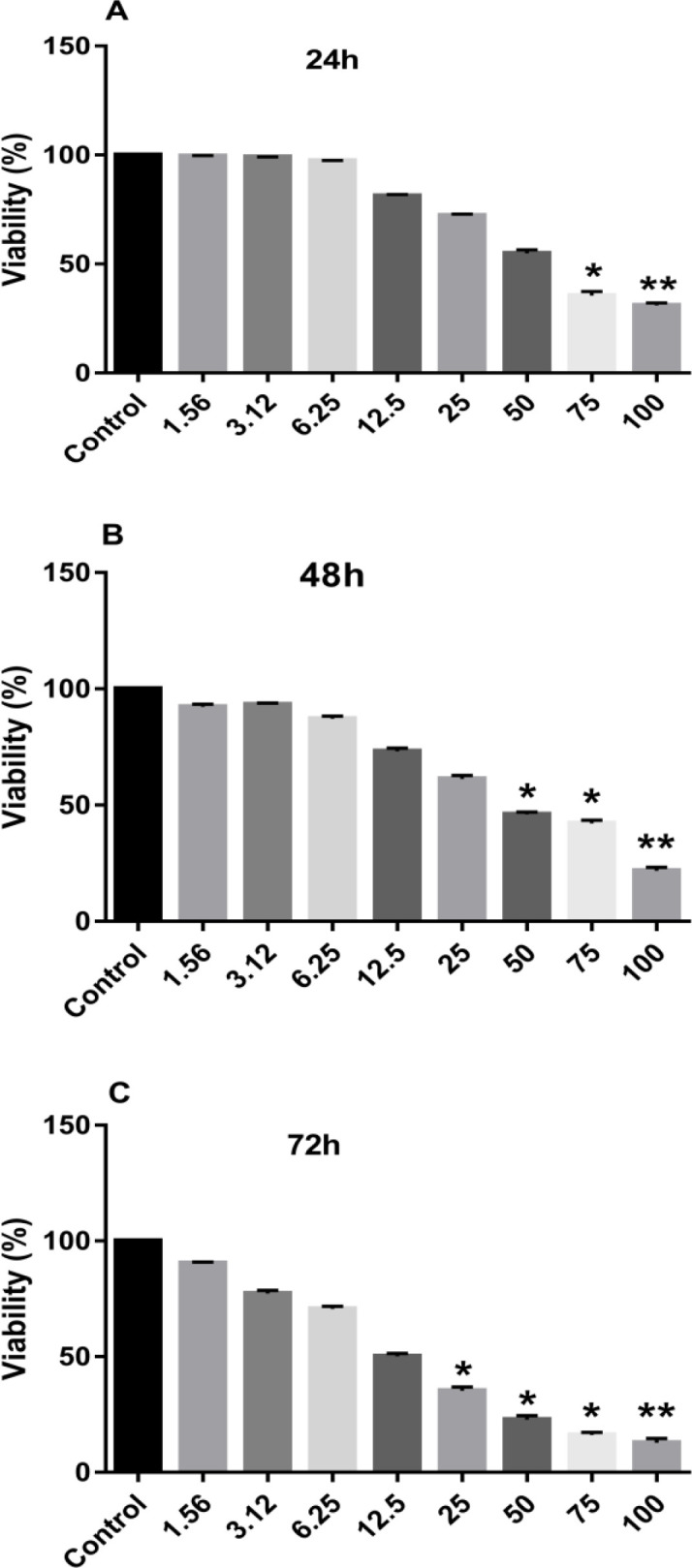
Cytotoxic effects of britannin on MCF-7 cell line. The cells were treated with various concentrations of britannin (0, 1.56, 3.12, 6.25, 12.5, 25, 50, 75, and 100 µM) for 24 (A), 48 (B), and 72 (C) hr. The MTS assay was performed to determine the viability of MCF-7 cells. The data are expressed as mean±SD of three independent experiments. Significant differences were found between the treatment and control groups at *p<0.05 and **p<0.01.

Treating MCF-7 breast cancer cells with increasing concentrations of britannin (0-100 µM) showed the anti-viability impact of this natural compound on these cancer cells. This reduction in cell proliferation during 24 hr was significantly shown at concentrations of 75 and 100 µM when compared with the control cell line (Figure 1A). Furthermore, treatment of MCF-7 cells with the mentioned concentrations of britannin for 48 and 72 hr caused a marked decline in cell proliferation at concentrations of more than 50 and 25 µM, respectively (Figures 1B and C). IC50 values for 24, 48, and 72 hr of britannin treatments were calculated as 51.65, 48.46, and 14.13 µM, respectively. Therefore, all other experiments were performed using IC25 concentration of britannin (25 µM) in a 48 hr treatment period.

**Effect of britannin on apoptosis markers in MCF-7 cells**

**Figure 2:**
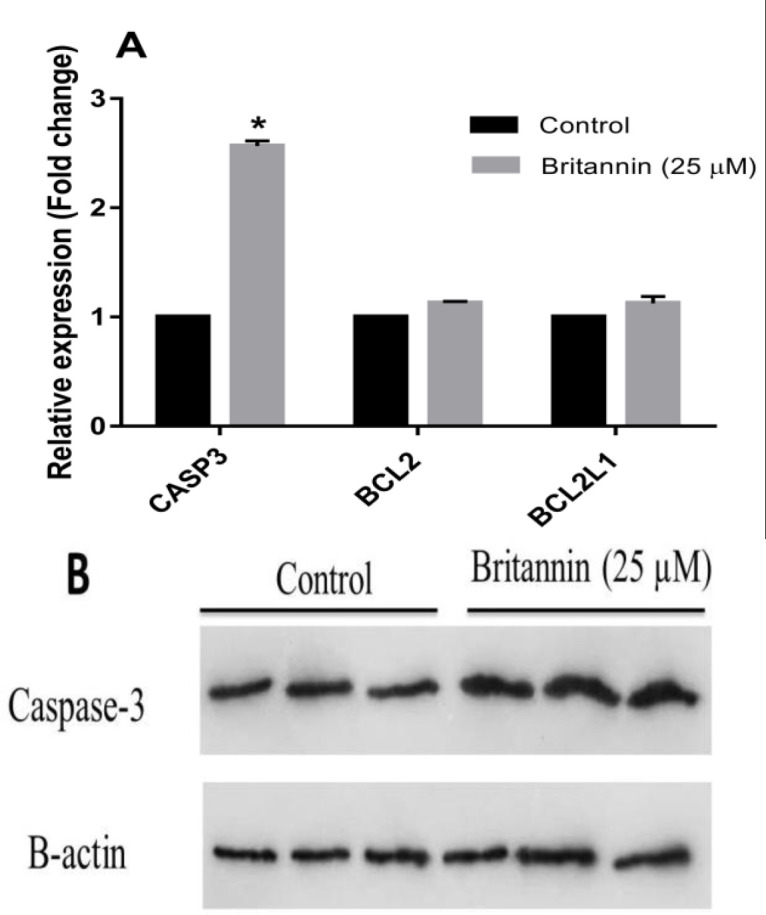
Effect of britannin treatment at the concentration of 25 µM for 48 hr on the expression of some apoptosis markers in MCF-7 cell line. (A) Real-time PCR analysis of the expression of apoptosis-related genes including *CASP3*, *BCL-2*, and *BCL2L1* after exposure of MCF-7 cells to britannin. (B) Effect of britannin on the protein expression of caspase-3. The data are presented as means±SD for the three independent experiments (*p<0.05, compared with the control).

As described earlier, treatment of MCF-7 cells with britannin at a concentration of 25 µM (IC25) for 48 hr was performed to weigh its impacts on other parameters. Thus, in the next step, the MCF-7 cells were exposed to 25 µM britannin for 48 hr to weigh its impacts on apoptosis markers, including *Bcl2*, and *BCL-xL/BCL2L1* at mRNA and caspase-3 at mRNA and protein levels. The results uncovered that britannin at a concentration of 25 µM significantly induced apoptosis-promoting gene expression, caspase-3, but had no remarkable effect on the expression levels of two anti-apoptotic genes *Bcl2*, and *BCL-xL/BCL2L1* (Fig 2A). Based on these data, western blotting was performed for the caspase-3 enzyme to affirm the effect of britannin on its protein level. As shown in Figure 2B, treating the MCF-7 cell line with 25 µM britannin for 48 hr markedly upregulated levels of caspase-3 protein.

**Effect of britannin on JAK/STAT pathway in MCF-7 cells**

**Figure 3:**
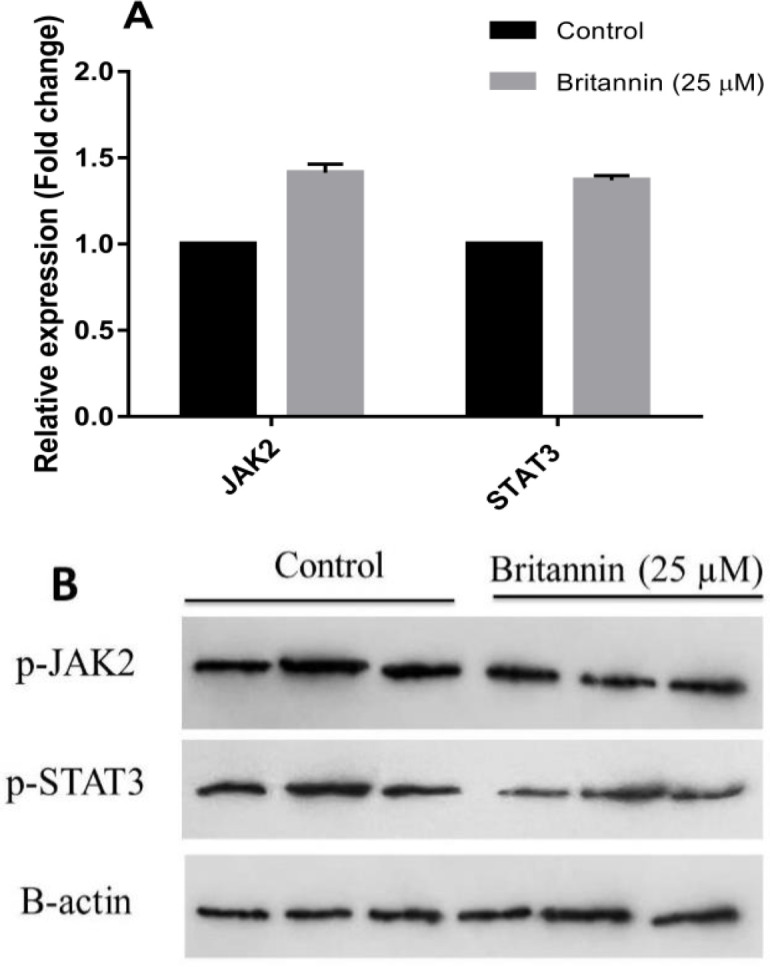
Effect of britannin (25 μM, 48 hr) treatment on the gene levels of *JAK2* and *STAT3*. (B) Effect of britannin (25 μM, 48 hr) on the levels of phosphorylated and activated forms of JAK2 and STAT3 proteins. p-values less than 0.05 were considered significant compared with the control.

To evaluate the probable effect of britannin on the function and activation of the JAK/STAT pathway in MCF-7 cells, they were treated with the compound at a concentration of 25 µM for 48 hr. Afterward, the levels of JAK2 and STAT3 at mRNA and protein levels were determined using real time-PCR and western blotting. As depicted in Figures 3A and B, britannin exerted no significant effect on the expression of both *JAK2* and *STAT3* genes. However, it significantly downregulated the phosphorylated and activated forms of JAK2 and STAT3 in MCF-7 cells. This suggests that britannin can act as a potential inhibitor of the JAK/STAT pathway in MCF-7 cells.

**Effect of britannin on autophagy markers in MCF-7 cells**

**Figure 4:**
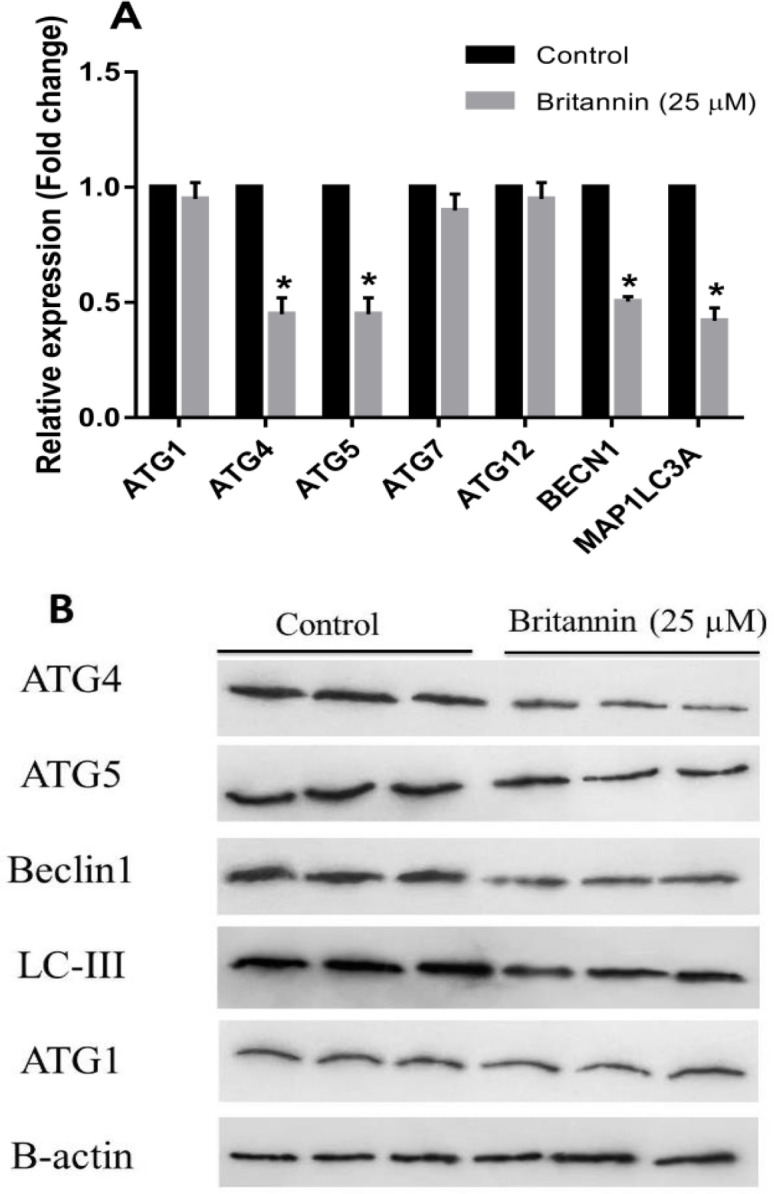
(A) Effect of britannin treatment at the concentration of 25 µM for 48 hr on the expression of autophagy-related markers at mRNA levels in MCF-7 cell line. (B) Effect of britannin treatment (25 µM for 48 hr) on the expression of autophagy markers in MCF-7 cell line at protein levels. The data represent means±SD for the three separate experiments (*p<0.05, compared with the control).

In the final step, MCF-7 cells received 25 µM britannin for 48 hr to evaluate the effect of this natural compound on the expression of autophagy-related markers in these cells. Based on our results, britannin at the dose of 25 µM significantly diminished four key autophagy markers, including *ATG4*, *ATG5*, *BECN1*, and *MAP1LC3A* at mRNA levels in the MCF-7 cell line. However, the compound had no remarkable effect on the transcripts of *ATG1*, *ATG7*, and *ATG12*. The results of western blotting also confirmed that britannin could not affect the expression of ATG1 protein, however it significantly prohibited the expression of ATG4, ATG5, Beclin1, and LCIII proteins in the MCF-7 cell line when comparing with the control (Figure 4A and B). These data demonstrated that britannin may be involved in the blockade of autophagy in MCF-7 cells.

## Discussion

The development of resistance to current therapies for breast cancer urges the need to find new remedies to cure patients with the disease (Malik et al., 2022). Programmed cell deaths have been established as promising molecular targets among different ways of cancer therapy (Maniam, 2021). In a previous study, we demonstrated that britannin can act as an apoptosis inducer in two metabolically different forms of breast cancer cell lines, MDA-MB-468 and MCF-7 (Hamzeloo-Moghadam et al., 2015). Hence, the present exploration aimed to assess the influence of britannin on the expression of some apoptosis and autophagy markers in the MCF-7 cell line. Furthermore, the effect of britannin on JAK/STAT signaling, as one of the pathways involved in the apoptosis of cancer cells, was examined.

In the present study, increasing concentrations of britannin (0-100 µM) caused a decline in the viability of MCF-7 breast cancer cells in a dose- and time-dependent manner. Treating these cells with britannin for 24 hr showed a significant anti-proliferative effect at concentrations of more than 75 µM. Britannin treatment for 48 and 72 hr could suppress the viability of MCF-7 cells at concentrations of more than 50 and 25 µM, respectively. IC50 values of britannin treatment for 24, 48, and 72 hr were calculated as 51.65, 48.46, and 14.13 µM, respectively. Based on these IC50 values, britannin treatment of the cells for the molecular tests was performed using IC25 concentration of the drug (25 µM) for 48 hr. These data are in accordance with our previous explorations on the anti-cancer effects of britannin. This natural compound has been found to decrease the viability of acute lymphoblastic leukemia cells, MOLT-4, with no remarkable cytotoxic effects on normal cells (Mohammadlou et al., 2022b). It also exerted significant cytostatic effects on several cancer cell lines (Moghadam et al., 2012). The present data confirmed that britannin exerts its cytotoxic effects on cancer cells by inducing apoptotic cell deaths. Notably, treating the MCF-7 cells with britannin at the concentration of 25 µM significantly elevated the expression of caspase-3, as an executioner enzyme in apoptosis, with no considerable effect on the expression of anti-apoptotic *Bcl2*, and *BCL-xL/BCL2L1* genes. This affirms that britannin could act as an apoptosis-inducing factor in breast cancer cells. Hamzeloo-Moghadam et al also evidenced that britannin amplified the activity of caspase-3 and -9 in MCF-7 and MDA-MB-468 cells. However, their data revealed downregulated Bcl-2 and upregulated Bax (Hamzeloo-Moghadam et al., 2015). They suggested that britannin induced apoptosis in these cancer cells by a mechanism that implicates the mitochondrial signaling pathway. Moeinifard et al obtained a similar set of data using britannin treatment of human pancreatic cancer cells (Moeinifard et al., 2017). However, they asserted that the mechanism of britannin action in these cells implicates the AKT-FOXO1 pathway probably in a reactive oxygen species (ROS)-dependent manner. Mohammadlou et al also affirmed that britannin stimulated apoptosis in acute lymphoblastic leukemia cell lines in a ROS-mediated manner (Mohammadlou et al., 2021). In another study by this team, they treated both chronic and acute myeloid leukemia cells with britannin and reported that the compound triggered apoptosis in both cell lines by acting on anti- and pro-apoptotic factors through a mechanism that involves the p21/p27 signaling pathway (Mohammadlou et al., 2022a). In the current exploration, we showed that britannin stimulated apoptosis in the MCF-7 cell line by a mechanism that hampers JAK2/STAT3 signaling pathway. Our data indicated that britannin treatment of MCF-7 cells did not affect the total expression of JAK2 and STAT3. However, it prohibited the phosphorylation and activation of these two factors in these cancer cells. As reported in the literature, targeting JAK/STAT signaling by inhibiting the activation of this pathway is an efficient and promising way to cease tumor growth by inducing apoptotic cell death in tumor cells (Liu et al., 2018; Rajabi et al., 2020). 

In the final step, the effect of britannin on autophagic cell death was examined. Accordingly, MCF-7 cells received britannin and the expression of several key autophagy markers including ATG1, ATG4, ATG5, ATG7, ATG12, Beclin1, and LCIII was measured. The results revealed that britannin decreased the gene and protein expression of ATG4, ATG5, Beclin1, and LCIII in MCF-7 cells in comparison to the control. This may suggest the inhibitory role of britannin in autophagic cell death in the MCF-7 cell line. According to the literature, apoptosis and autophagy function on opposite sides in cancerous cells (Buzharevski et al., 2019; Xie et al., 2020). In fact, autophagy plays a dual role in some cancer cells. For example, it can inhibit breast cancer cell growth, but protects it by limiting and delaying its death in response to stressors (Abedin et al., 2007). One study showed that autophagy can act as a mechanism for the development of tamoxifen resistance in breast cancer cells, and consequently suggested the inhibition of autophagy to resensitize these cells to the drug (Ding et al., 2019). Therefore, the outputs of the current investigation may suggest britannin as a potential autophagy inhibitor to be used in combination therapies against drug-resistant breast cancer. Choi et al reported that the blockage of autophagy leads to the enhancement of apoptosis in gastric cancer cells (Choi et al., 2015). Li et al also identified that the blockage of autophagy could sensitize MCF-7 cells to an apoptosis inducer known as cyclic tetrapeptide CTS203 (Wang et al., 2015). However, autophagy regulation appears to be context-dependent. For example, Cui et al provided evidence to show that britannin simultaneously induced both autophagy and apoptosis in liver cancer cells by suppressing mammalian target of rapamycin (mTOR) and activating AMP-activated protein kinase (AMPK) in a ROS-associated way (Cui et al., 2018). 

Looking at the current data, it is implied that britannin induces apoptosis in MCF-7 breast cancer cells by a mechanism that involves the inhibition of the JAK/STAT signaling pathway. Our findings also unraveled that the cell death-inducing activity of britannin in MCF-7 cells is not only exerted by stimulating apoptosis but it also hampers autophagy in these cancer cells. As mentioned before, inhibiting autophagy can hamper drug resistance in cancer cells. Therefore, britannin, as a potential autophagy inhibitor, can be used as an adjutant therapeutic to enhance the effects of anticancer drugs. This may suggest britannin as a novel and efficient drug for the suppression of breast tumors.

## Conflicts of interest

The authors have declared that there is no conflict of interest.
